# Global cooling as a driver of diversification in a major marine clade

**DOI:** 10.1038/ncomms13003

**Published:** 2016-10-04

**Authors:** Katie E. Davis, Jon Hill, Tim I. Astrop, Matthew A. Wills

**Affiliations:** 1 Milner Centre for Evolution, Department of Biology and Biochemistry, The University of Bath, Bath BA2 7AY, UK; 2 Environment Department, University of York, York YO10 5NG, UK

## Abstract

Climate is a strong driver of global diversity and will become increasingly important as human influences drive temperature changes at unprecedented rates. Here we investigate diversification and speciation trends within a diverse group of aquatic crustaceans, the Anomura. We use a phylogenetic framework to demonstrate that speciation rate is correlated with global cooling across the entire tree, in contrast to previous studies. Additionally, we find that marine clades continue to show evidence of increased speciation rates with cooler global temperatures, while the single freshwater clade shows the opposite trend with speciation rates positively correlated to global warming. Our findings suggest that both global cooling and warming lead to diversification and that habitat plays a role in the responses of species to climate change. These results have important implications for our understanding of how extant biota respond to ongoing climate change and are of particular importance for conservation planning of marine ecosystems.

## Abstract (web summary)

In many groups of organisms, speciation rates are higher when global temperatures are warmer. Here, Davis *et al*. find that marine crustaceans in the Anomura clade have higher speciation rates during cooler periods, whereas their freshwater relatives have the more typical relationship of higher speciation rates in warmer periods.

## Graphical abstract

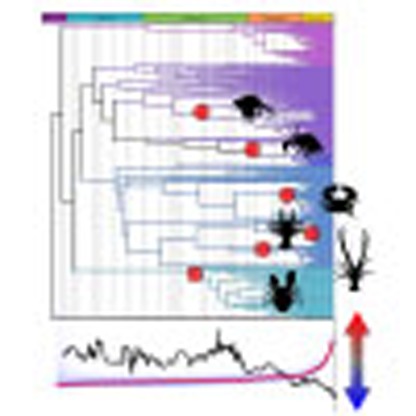

Changes in climate, particularly global mean temperatures, have long been identified as drivers of diversity turnover on macroevolutionary scales^1^,^2^,^3^,^4^. Current rates of extinction, partly resulting from anthropogenic climate change^5^, are rapidly approaching those seen in the ‘big five' extinction events of the geological past, with the present biodiversity crisis now being heralded as the sixth^6^. However, to predict the probable effects of current and future climate change it is vital that we understand past processes and trends. Of particular concern is the threat to our oceans and freshwater ecosystems^7^. The oceans contain an estimated 25% of extant global biodiversity^8^, and marine organisms provide a vital contribution to global ecology^9^. However, while the responses of shelf marine invertebrates, as a whole, to a variety of climatic and other factors has been investigated at a taxic level, this investigation has yielded contradictory results; some studies found that warming induced increased diversification, while others found the opposite effect^10^,^11^,^12^. By contrast, more focussed studies on marine molluscs over shorter time scales have found no relationship between diversification and environmental factors. Rather, species either track habitat^13^ or nutrient availability^14^, or they go extinct. Terrestrial vertebrates, on the other hand, appear to show a positive correlation between speciation and global temperature^15^,^16^,^17^, while marine vertebrates show either a positive correlation with warming or no correlation with climate^16^,^17^,^18^. To date, no work has been carried out on the relationship between speciation and climate of marine invertebrates within a phylogenetic framework.

Here we examine the relationship between climate and biological diversification for Anomura (hermit crabs, king crabs, porcelain crabs, mole crabs and squat lobsters). Critically, we do this in a phylogenetic framework, using the largest tree ever assembled for the group. Anomura are an enigmatic infraorder, containing species with a diverse array of ecologies and encompassing a particularly impressive disparity of forms^19^. They are also remarkable in having independently evolved carcinisation in multiple lineages^20^. Anomura are united by the general synapomorphy of a reduced anterior-most pair of pereiopods used in gill cleaning, and the absence or severe reduction of the tail. To date, 2,500 extant species have been described^21^,^22^. The fossil record contains representatives of nearly all extant families and the infraorder spans the Late Triassic (Norian/Rhaetian) to the present day (ref. 23). Anomura have colonized deep marine (from depths of over 5,000 m) to littoral environments, and are also found in freshwater and semi-terrestrial habitats. Historical taxonomic controversies are now largely resolved^19^, therefore their evolution provides an excellent system in which to investigate the relationship between climate and speciation in aquatic invertebrates.

Previous studies investigating the link between climate and biological diversification have largely used a taxic approach, treating species, genera and families as independent and assessing changes in taxonomic diversity through time. Using a time-calibrated phylogenetic supertree of Anomura, which broadly matches a recent molecular phylogeny and is consistent with recent taxonomic revisions, we calculate diversification rates for the history of Anomura. We then perform correlation analyses of palaeo-temperature proxies, and these diversification rates using a Bayesian framework. Our analyses show a negative correlation between the whole tree speciation rates and palaeo-temperature. In detail, marine taxa show the same negative correlation, but the single freshwater clade shows the opposite relationship: increased speciation with warming. These results suggest that habitat plays a role in how speciation rates are affected by temperature changes, which in turn has implications for how we manage and conserve extant aquatic biota.

## Results

### Supertree construction

**Figure 1:**
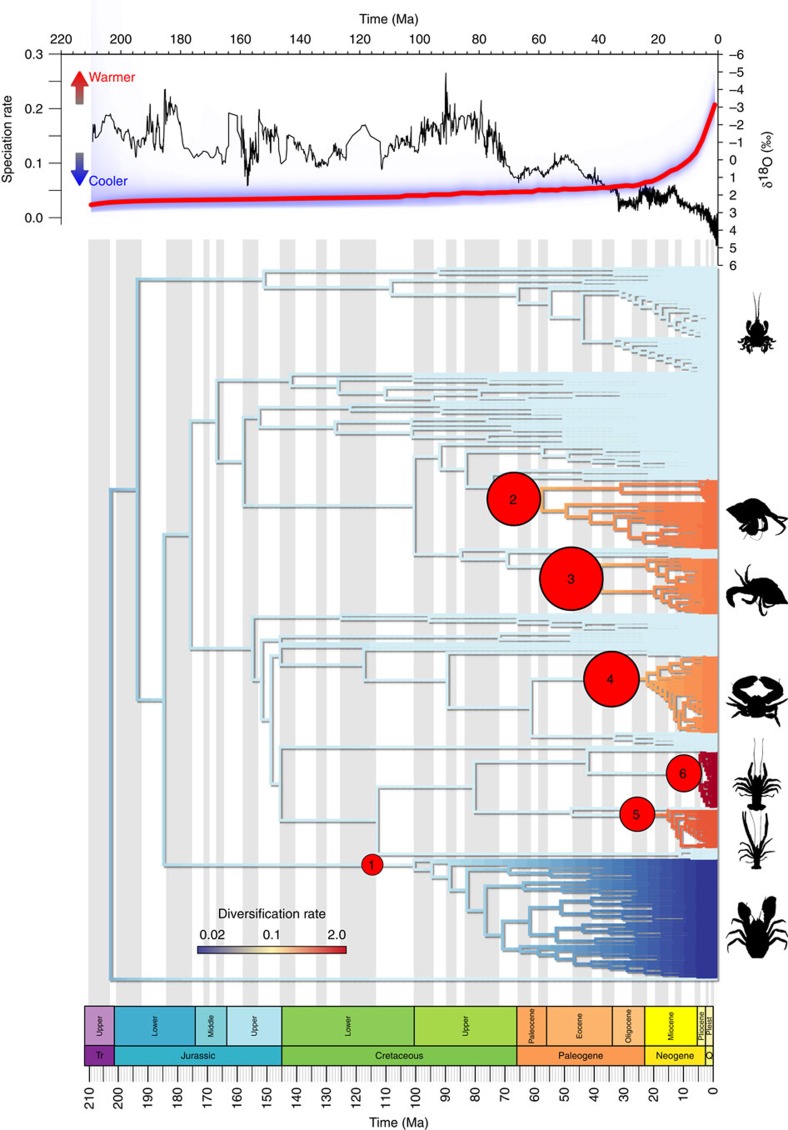
Phylogeny of Anomura. The phylogeny is coloured to show diversification rate (dark blue: low rate; red: high rates) and scaled to the geological timescale (bottom)^54^. The top panel shows the δ^18^O curve^30^,^31^ superimposed on the average speciation rate for the anomuran phylogeny. Significant diversification rate shifts are shown by red circles. Larger circles indicate higher probabilities. Highlighted clades are (from top to bottom) are Hippoidea, Paguridae+Lithodoidea, Diogenidae+Coenobitidae, Porcellanidae, *Paramunida*, *Munida* and Aegloidea. Diversification rate shifts are labelled chronologically from 1 to 6. See text for details.

We constructed a phylogenetic supertree^24^,^25^ using 60 source trees from 40 papers published between 1986 and 2011. The resulting tree of 372 taxa is the largest phylogeny of Anomura published to date (Fig. 1). The overall structure of the supertree is broadly similar to that in a recent molecular phylogeny (137 taxa) of Anomura^19^, and is also consistent with recent taxonomic revisions^26^, notably a much reduced Galatheoidea (Supplementary Fig. 1). In our tree all superfamilies are recovered as monophyletic with the exception of the Paguroidea. Hippoidea are basalmost, followed by Aegloidea+Lomisoidea. The Chirostyloidea+Galathoidea form a sister clade to the Paguroidea which contains the Lithodoidea. The resolution of Lithodoidea within Paguroidea is well-supported by both molecular and morphological data^27^. The supertree was time-calibrated using fossil age data. Three additional trees were generated using random perturbations of the fossil age data to simulate changes to node calibrations that could affect the robustness of our findings.

### Diversification analysis

**Figure 2:**
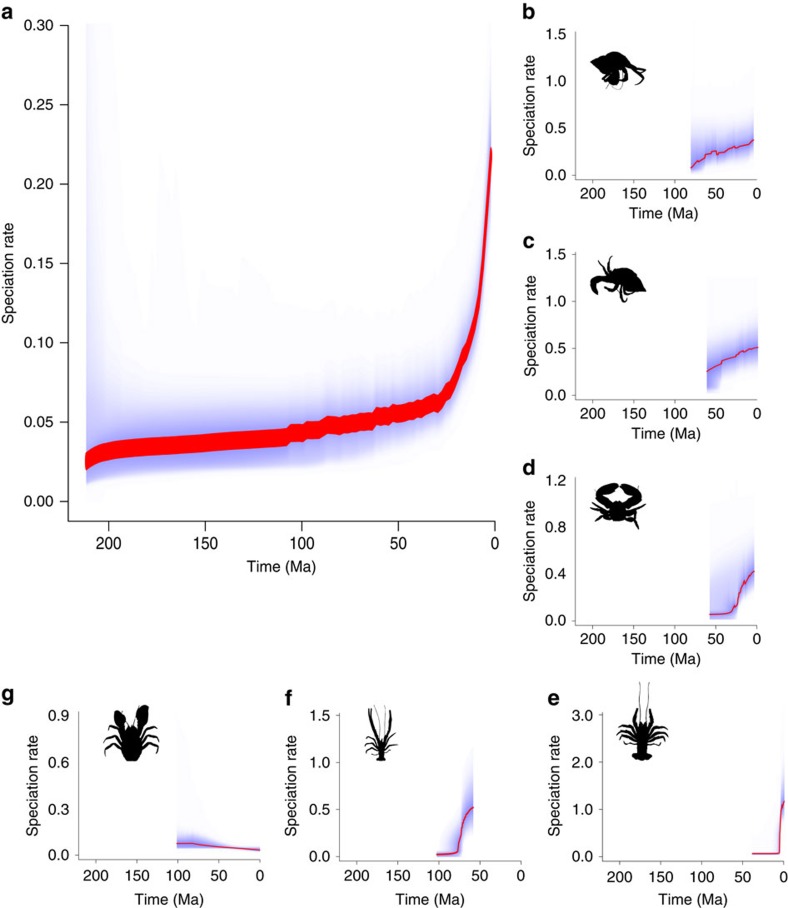
Speciation rates of Anomura and clades. The large panel (**a**) shows the speciation rate for the whole tree with the inset histogram showing the distribution for all recorded speciation rate curves for both Pearsons's and DCCA correlation coefficients. The mean correlation for the whole tree is −0.709. Additional panels (**b**–**g**) for each of the clades that show a significant diversification rate shift. Clades are (from top right to bottom left): (**b**) Paguridae+Lithodoidea (clade 2 in Fig. 1), (**c**) Diogenidae+Coenobitidae (clade 3 in Fig. 1), (**d**) Porcellanidae (clade 4 in Fig. 1), (**e**) *Paramunida* (clade 6 in Fig. 1), (**f**) *Munida* (clade 5 in Fig. 1) and (**g**) Aegloidea (clade 1 in Fig. 1).

Using the phylogeny described above we calculated diversification rates via the BAMM framework, which implements a Metropolis Coupled Monte Carlo (MCMC) method to calculate diversification rates along lineages^28^. We accounted for sampling bias in the phylogeny for the diversification analysis by providing BAMM with a list of species-specific sampling probabilities. Species lists and classifications used to calculate the taxon sampling were obtained from WoRMS^29^. The analysis identified six significant rate shifts occurring at different times and within six distinct clades (Figs 1 and 2). Changes in diversity dynamics are therefore not simply triggered coincidentally and in parallel across multiple lineages (as might be expected for some universal environmental driver), but rather occur at different times contingent on the clade. All of the rate shifts occur in the last ∼100 Myr with three in the last 50 Myr, despite the Triassic origination of Anomura. Shifts are therefore more numerous and more recent than found in previous, less speciose studies^19^. The additional trees obtained using the altered node calibrations found the same diversification shifts, plus an additional shift with the origination of the Coenobitidae in the Miocene.

### Climate correlation

**Figure 3:**
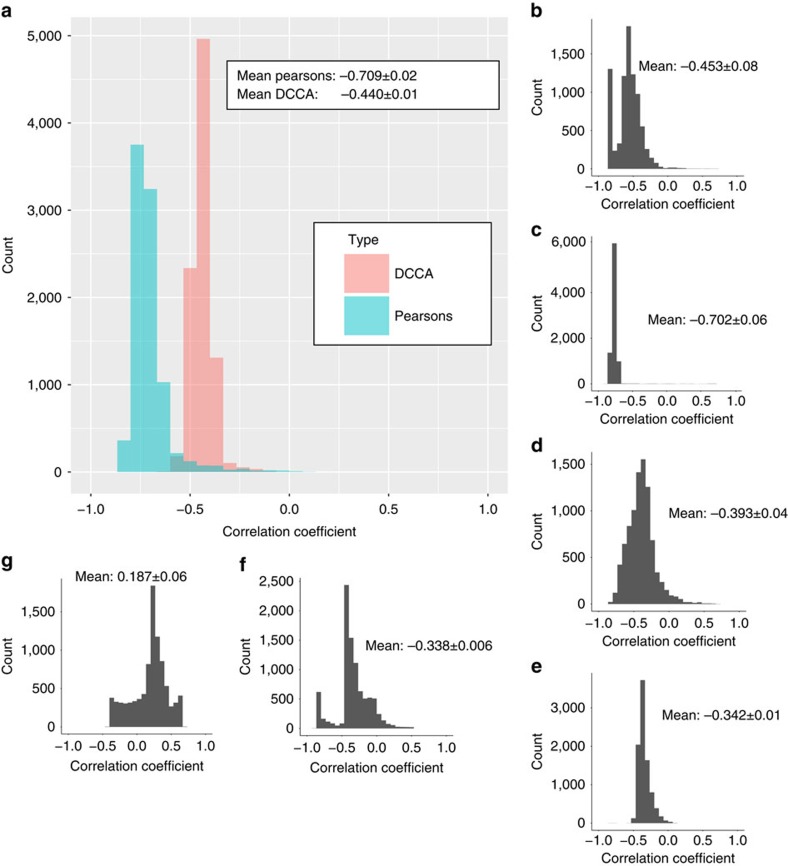
Correlation coefficients of speciation rate and palaeo-temperature. Each panel shows a histogram of correlation coefficients between paleao-temperature and the 9,000 diversification analyses. Panel (**a**) shows the result of analyzing the whole tree using both Pearson's R and DCCA (see methods) with a mean correlation coefficient of −0.709. Additional panels (**b**–**g**) show the results of the DCCA correlation for each clade that showed a significant rate shift, with the mean correlation and an estimate of the error. Clades are (from top right to bottom left): (**b**) Paguridae+Lithodoidea (clade 2 in Fig. 1), (**c**) Diogenidae+Coenobitidae (clade 3 in Fig. 1), (**d**) Porcellanidae (clade 4 in Fig. 1), (**e**) *Paramunida* (clade 6 in Fig. 1), (**f**) *Munida* (clade 5 in Fig. 1) and (**g**) Aegloidea (clade 1 in Fig. 1).

Using these diversification rate shifts to identify clades of interest we found speciation rates to be strongly correlated with global palaeo-temperature as identified from oxygen isotope (δ^18^O) records, used as a proxy for palaeo-temperature^30^,^31^ (Fig. 1). We performed correlations of all realizations of the diversification curve in the MCMC analysis (9,000 in total) for all Anomura against the global δ^18^O curve. For each realization a correlation coefficient was calculated between −1 (speciation rate increases with cooler temperatures) and 1 (speciation rate increases with warmer temperature). If the correlation coefficient is zero then temperature has no effect on speciation rate. We then tested whether the distribution of all 9,000 correlation coefficients differed from the null hypothesis of a zero mean correlation coefficient (that is, no temperature correlation). Considering all Anomura together resulted in a significant negative mean correlation (detrended cross-correlation analysis, ρ_DCCA_(s)=−0.44, *P*<2e–16) with very few simulations finding a positive correlation (Fig. 3). This finding implies that over the entire Anomura, rates of speciation increase with cooler temperatures. We repeated this within all of the clades that showed a significant diversification rate shift. This revealed that marine clades follow this same pattern, while the freshwater clade (Aegloidea) shows the opposite trend: speciating more rapidly with warmer temperatures (Table 1).

The earliest diversification rate shift (Fig. 1, shift 1) occurs in the Upper Cretaceous and is located basally within the Aegloidea (freshwater anomurans). The correlation of aeglid speciation rates against global δ^18^O is 0.19 (detrended cross-correlation analysis, *P*<2e^−16^, Fig. 3) indicating that higher global temperatures were a potential causal factor of the increased speciation rates. This rate shift is also associated with a significant habitat change as the aeglids begin to colonize mid-latitude freshwater habitats in South America^19^. In the Late Cretaceous we see diversification rate shifts in Diogenidae (Fig. 1, shift 3) and in the clade containing Paguridae+Lithodoidea (both clades of hermit crabs—Fig. 1, shift 2). Both show strong negative correlations between temperature and speciation rate of −0.45 and −0.70 respectively (detrended cross-correlation analysis, *P*<2e^−16^, Fig. 3) indicative of cooler temperatures driving speciation. Porcelain crabs (Porcellanidae, Fig. 1, shift 4) show a further negative correlation between speciation rate and temperature (detrended cross-correlation analysis, ρ_DCCA_(s)=−0.39, *P*<2e^−16^). A rate shift also occurs in the Oligocene in *Paramunida* (squat lobsters, Fig. 1, shift 5), which show a small negative correlation of speciation rate to temperature (detrended cross-correlation analysis, ρ_DCCA_(s)=−0.34, *P*<2e^−16^). During the Miocene we see another negative correlation (detrended cross-correlation analysis, ρ_DCCA_(s)=−0.34, *P*<2e^−16^,) of speciation rate and temperature in *Munida* (squat lobsters, Fig. 1, shift 6). This is the most recent rate shift, occurring at just 16 Ma. In addition to these correlations, we see another diversification rate shift in Coenobitidae occurring in the additional trees with subtly altered node dates generated to account for uncertainty in the data. Coenobitidae are the only anomurans to have adopted a semi-terrestrial mode of life therefore, as in the case of the aeglids, this shift occurs coincident with a change in habitat. This diversification rate shift also shows a negative correlation with temperature (detrended cross-correlation analysis, ρ_DCCA_(s)=−0.34 to −0.56, *P*<2e^−16^).

## Discussion

At the infra-ordinal level, Anomura demonstrate increased speciation with cooler temperatures; all clades that undergo a significant shift in diversification rate also show this relationship with the exception of the freshwater aeglids in which the pattern is reversed. The process by which global cooling results in an increase in diversification in some marine fauna could be due to a number of factors^32^ including tectonic activity, changes in sea level, decreased hypoxia or changes to ocean currents. Tectonic activity has been linked to fluctuations in evolutionary rates^33^, and during the last 60 Ma there has been extensive tectonic activity associated with the opening of the Atlantic ocean^34^, which coincides with four of the six diversification rate shifts identified in this study. There is a significant link between tectonic activity and climate^35^, which makes it difficult to extract the primary driver. However, tectonics alone is unlikely to be the direct driver of speciation due to the global distribution of clades that show diversification shifts. Changes in speciation rate cannot, therefore, be attributable to tectonic events, such as the opening of the North Atlantic, despite the apparent correlation of timing. Global cooling also results in the lowering of sea level as seawater is sequestered into ice sheets^36^; this reduces shallow-shelf habitats causing habitat fragmentation and thereby increasing the potential for allopatric speciation. Marine anomurans are predominantly, though not exclusively, shallow water dwellers and therefore particularly susceptible to marine regression. Bodies of freshwater would not be affected in this manner by sea level regression, which may explain the observed correlation of increased diversification and global warming in the freshwater anomurans (aeglids) that remains consistent with previous work^10^,^11^. Moreover, higher temperatures have a tendency to increase hypoxia, which is problematic for benthic species^37^, such that a greater area of suitable habitat will be available during cooler periods. Finally temperature change also drives changes in ocean currents^38^, which may open up new habitats for marine taxa with planktonic larval stages, and may also lead to changes in heat distribution in the oceans. In addition to external factors, niche modification from invasion of species may be also be a driver of marine invertebrate speciation rates over geological time scales^39^, and species may track habitats during times of climatic variation^13^.

The effects of global temperature change are therefore tightly linked and difficult to tease apart. It is likely that multiple mechanisms drive the relationship between decreasing temperature and increasing speciation rates; a pattern that we speculate may extend to other groups of shallow-shelf dwelling invertebrates. This stands in contrast to patterns observed in terrestrial and marine vertebrates, whereby periods of warming (not cooling) elicit diversification^10^,^40^. Hence, the relationship between global temperatures and the dynamics of faunal turnover are complex and the response of any given species to climate change may be strongly contingent upon its habitat and mode of life. While a model that simply predicts declining speciation rates with global warming would be attractive and its implications clear, that is, increased warming will accelerate the rates of anthropogenic diversity decline already caused by other mechanisms, the reality is more complex. Given that crustaceans play a crucial role in marine ecosystems, as well as providing an important food source for many societies, it is important that management of marine ecosystems accounts for this potential loss in biodiversity. Habitat clearly plays a role in species response to climate change, as demonstrated here, and future work will need to consider this when attempting to predict future speciation rates and how to conserve key species. The ultimate goal should be more realistic models of diversity dynamics that utilize information on clade specific responses to multiple factors. These have great potential for better predictions of the probable fate of organisms in wake of future climate changes^41^. With the current state of knowledge, however, limiting warming and human influence on the environment is still the best way of preventing species extinction.

## Methods

### Data collection and processing

Source trees were identified using the Web of Knowledge Science Citation Index^42^ with the search terms: phylog*, taxonom*, systematic* and clad* in conjunction with all scientific and common names for Anomura from infraorder to superfamily level. These searches were carried out for the years 1980–2011, and all papers potentially containing phylogenetic trees were examined. All source trees, along with associated meta-data, such as bibliographic information, character data and optimization criteria used, were recorded. See Supplementary Table 1 and Supplementary Data 1 for data used.

We followed the protocol previously described^43^ in which source trees needed to meet several criteria for inclusion in the analysis. These criteria were: (1) it should be explicit that the author's intention was to construct a phylogeny, (2) the characters and taxa used in the analysis must be clearly identifiable and (3) the tree should be based on an analysis of a novel, independent dataset. Non-independence was defined as two or more studies that used the same character data and had either identical taxa, or alternatively where one taxon set was a subset of the other. In this latter case, the less comprehensive tree was removed from the dataset. In the former case trees were combined into a summary consensus tree to create a single tree for inclusion in the supertree analysis.

### Nomenclature and taxonomic consistency

Operational taxonomic units (OTUs) were standardized to avoid the inclusion of higher taxa and vernacular names that would artificially inflate the number of taxa in the analysis. In addition synonyms and misspellings were corrected as otherwise this could lead to inconsistencies. Names were standardized using the online WoRMS database^29^. Paraphyletic taxa were dealt with using the STK (refs 24, 25), by calculating all possible positions of paraphyletic taxa in a source tree and building a mini-supertree from these. Higher taxa and vernacular names were removed from source trees by either substituting the constituent taxa of those groups into a polytomy or, where possible, substituting the actual species that the authors intended to represent. Definitions for higher taxa were adopted from WoRMS. This substitution stage did not introduce any taxa that were not already present in the dataset as any inconsistencies were flagged by the STK. Once nomenclature had been standardized, we checked that source trees had sufficient taxonomic overlap, such that each source tree was required to have at least two taxa in common with a minimum of one other source tree.

### Supertree construction

The most commonly implemented supertree method is Matrix Representation with Parsimony (MRP)^44^, whereby all taxa subtended by a given node in a source tree are scored as ‘1', taxa not subtended by that node are scored as ‘0', and taxa not present in that source tree are scored as ‘?'. Trees are rooted with a hypothetical, all-zero outgroup. MRP is currently the only supertree construction method with software implementation able to deal with large (100+ taxa) datasets. We used standard Baum and Ragan MRP coding^44^, and matrix creation was automated using the STK software^24^,^25^.

The matrix was analysed with TNT^45^ using the mult 30 option. A total of 1,000 replicates for each analysis were run, each using a different random starting point for the heuristic search. The intention of this method is to search as much of the tree space as possible within a reasonable computational time. The analysis found 432 MPTs of length 2,548 steps. Resolution was poor in both the strict and 50% majority rule consensus trees. We therefore computed a Maximum Agreement Subtree (MAST) using PAUP* (ref. 46) to remove the conflicting taxa. This reduced the number of ingroup taxa from 599 to 397. In addition, we also identified some rogue taxa in the resulting tree (see list in Supplementary Table 2), and these were removed from the final supertree^43^. The phenomenon of rogue taxa was first discussed by Bininda-Emonds and Bryant^47^ who noted that the MRP method can lead to the creation of spurious clades and relationships that are not present in any of the source trees (‘novel clades'). Although simulations have suggested that such anomalies are unlikely to be a significant problem^48^, empirical studies have found an incidence of novel clades affecting up to 3% of taxa^49^. The final tree is available in Supplementary Data 2.

### Phylogeny time-calibration

Supertree parsimony methods do not produce trees with meaningful branch lengths for inferring dates of relative splits, as any branch length data in the source trees is not retained by the MRP algorithm. Therefore, to construct a time-scaled phylogeny, we obtained fossil dates from fossilworks.org (Supplementary Data 3). Twenty-seven nodes were calibrated using fossil first occurrence data (see Supplementary Data 3 for further details). These fossils were assigned phylogenetically to either the stem or crown of clades using the taxonomy assigned in Fossilworks (Supplementary Data 4). Dated nodes were widely distributed throughout the tree and covered all major clades within Anomura. The R package ‘paleotree' (ref. 50) was used to scale the tree and extrapolate dates to the remaining nodes. To investigate the effects of subtle changes in the node dates and the potential subsequent effect on the tree, we re-ran a complete analysis on a number of scenarios in which the node date was shifted to a parent or child node at random. We generated new trees moving 10, 15 and 20% of dates in this manner. To calibrate the whole tree we chose to use the ‘equal' method, with a minimum branch length of 0.1 Myr. Estimates of rates and models of continuous trait evolution are sensitive to bias from the insertion of many short branch lengths^51^, hence the arbitrary variable used in other methods could have a significant effect on the diversification analyses performed subsequently.

### Diversification rate shift analysis

Diversification rates were assessed via BAMM, which uses an MCMC approach to calculate diversification rates and significant rate shifts. Four chains were executed for the analysis, each with a total of 20 million generations executed, with a minimum clade size of five taxa used to aid convergence. Ten thousand of the results were stored, with 1,000 discarded as ‘burn-in', leaving 9,000 samples for subsequent analysis with regards to temperature correlation. For details of the sampling and BAMM set-up see Supplementary Data 4 and 5. The analysis also accounted for non-complete coverage of taxa in the tree by specifying a clade-dependent sampling bias factor derived from the taxonomy in WoRMS^29^. Analyses without taking into account sampling yielded similar results. The diversification shifts were stable across a number of possible outcomes in the analysis with the top nine shift configurations showing the same rate shift, albeit at different probabilities (Supplementary Fig. 2). An additional shift may be present in Albuneidae, but this has a very low likelihood so is not considered further here. The most probable diversification rate shifts show remarkable consistency with regards to the branch location of each rate shift (Supplementary Fig. 3).

### Temperature correlations

Correlation of speciation rates with temperature utilized the output from BAMM by creating a speciation rate curve for the whole tree and for each clade that showed a significant diversification rate shift. These were then analysed using the script included in Supplementary Data 6. The δ^18^O data^30^,^31^ (Supplementary Data 7) were first smoothed using a Tukey running mean and then values linearly interpolated to the same time values available in the speciation rate data, which occurred in 0.1 Myr bins. For each of the 9,000 simulations stored from the BAMM analysis a linear correlation (Pearson's product moment correlation coefficient) analysis was performed and the results grouped. Given the normal distribution obtained, a Student's *t*-test was performed to assess the significance of the mean from zero. A zero mean would indicate that the BAMM simulations gave a non-significant correlation across the simulations. All simulations produced significant correlation values (*P*<0.05), with a normal distribution of results, and likewise all Student's *t*-tests produced significant results. However, the two time series used are autocorrelated at short lag times and hence may produce spurious results when using Pearson's correlation^52^. To investigate the effect of this, detrended cross-correlation analysis (DCCA) was used to account for the non-stationarity and autocorrelation^52^. Both analyses found the same relationship between speciation and global temperature for all clades. All analyses were carried out in R 3.2.2 (ref. 53) and the script used is included in Supplementary Data 6.

### Data availability

The authors declare that all data supporting the findings of this study are available within the article and its Supplementary Information files.

## Additional information

**How to cite this article:** Davis, K. E. *et al*. Global cooling as a driver of diversification in a major marine clade. *Nat. Commun.* **7**, 13003 doi: 10.1038/ncomms13003 (2016).

## Supplementary Material

Supplementary Information Supplementary Figures 1-3, Supplementary Tables 1-2, Supplementary References

Supplementary Data 1 Source trees used in the supertree construction (NEXUS formatted text file).

Supplementary Data 2 Final supertree used in subsequent analysis (NEXUS formatted text file).

Supplementary Data 3 Node dates used in dating tree. Node numbers correspond to the standard numbering in the ape R package (CSV file).

Supplementary Data 4 Set up file for BAMM used in all diversification analyses (ASCII text file).

Supplementary Data 5 Sampling file used in BAMM analysis. Each clade is given a fraction of the total number of known species compared to those in the tree (ASCII text file).

Supplementary Data 6 R script to carry out correlation analysis used Supplementary Data file 7 and the results from the diversification analysis (R script text file).

Supplementary Data 7 D18O data from references [30] and [31]. First column is the time (millions of years) and second column is the D180 value (CSV file).

## Figures and Tables

**Table 1:** Correlation coefficients of temperature proxy versus speciation rates for the clades highlighted in Fig. 1.

	Original tree		10% dates moved		15% dates moved		20% dates moved	
Clade number	PCC	DCCA	PCC	DCCA	PCC	DCCA	PCC	DCCA
Whole tree	−0.709	−0.440	−0.647	−0.405	−0.665	−0.411	−0.665	−0.409
1	**0.226**	**0.187**	**0.349**	**0.228**	**0.358**	**0.234**	**0.415**	**0.238**
2	**−**0.706	**−**0.453	**−**0.698	**−**0.657	**−**0.839	**−**0.776	**−**0.840	**−**0.778
3	**−**0.767	**−**0.702	**−**0.833	**−**0.767	**−**0.693	**−**0.656	**−**0.702	**−**0.663
4	**−**0.775	**−**0.39	**−**0.771	**−**0.388	**−**0.772	**−**0.385	**−**0.772	**−**0.383
5	**−**0.748	**−**0.338	**−**0.750	**−**0.345	**−**0.748	**−**0.337	**−**0.752	**−**0.351
6	**−**0.818	**−**0.342	**−**0.803	**−**0.607	**−**0.819	**−**0.344	**−**0.804	**−**0.607

Bold numbers indicate a positive correlation and are only found in the Aegloidea. The perturbed trees also show the same results.
