# Supplementary material for: Global cooling as a driver of diversification in a major marine clade
Source: Nat Commun. 2016 Oct 4;7:13003. doi: 10.1038/ncomms13003 (PMC5059450; doi:10.1038/ncomms13003)
Supplement: Supplementary Information — Supplementary Figures 1-3, Supplementary Tables 1-2, Supplementary References [file ncomms13003-s1.pdf]

Supplementary Figures

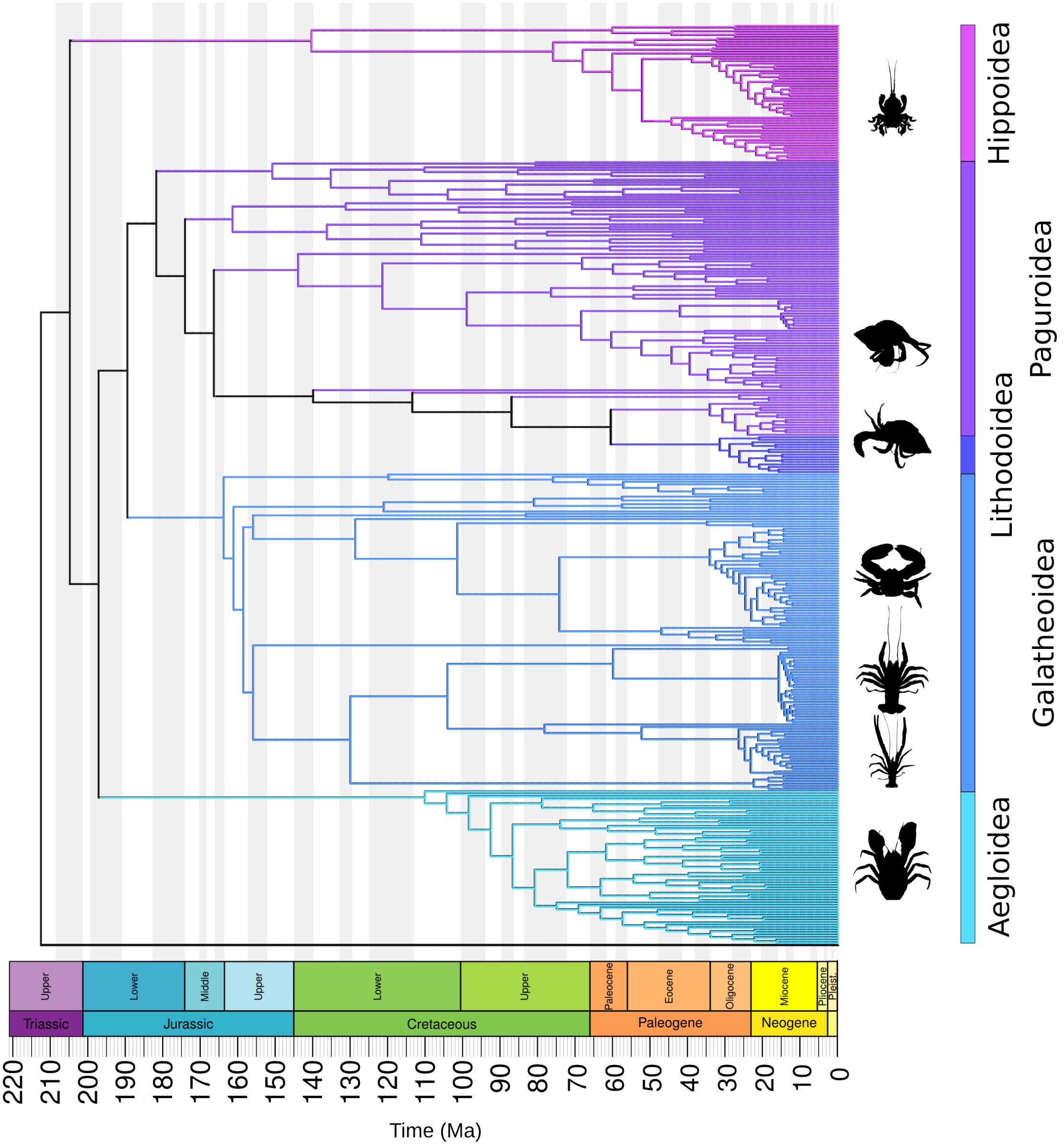

Supplementary Figure 1. Time-scaled phylogeny of Anomura, coloured to show superfamilies. This tree is the Maximum Agreement Subtree (MAST) computed from 432 MPTs of length 2548 in PAUP*[1]. The time-scale graphic was computed using strap[2] .

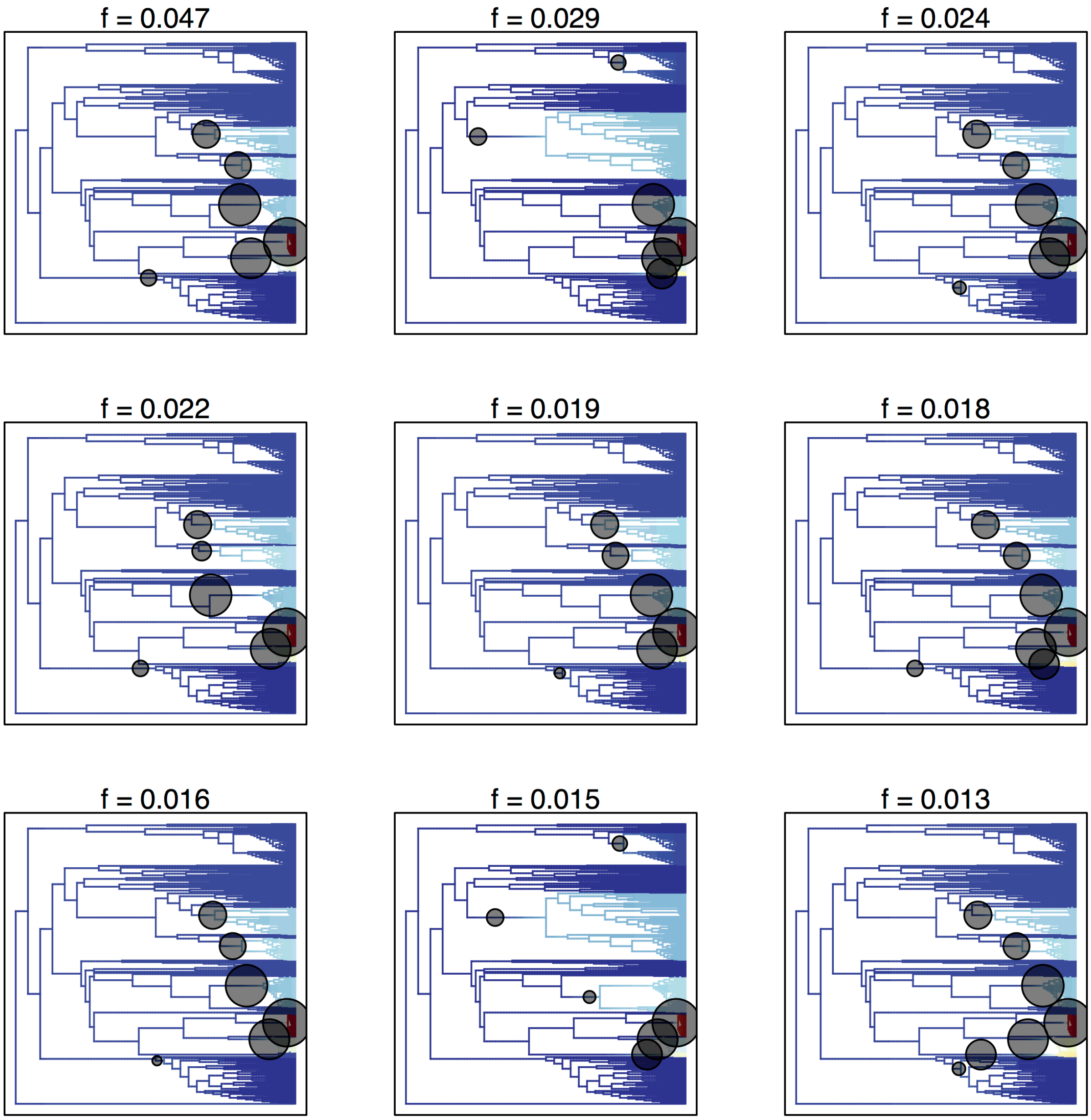

Supplementary Figure 2. Set of nine most probable shift configurations computed in BAMM[3]. These nine most probably configurations are remarkably stable, with little movement of the diversification shifts. Note that there is also a low probability of a shift within the superfamily Hippoidea as seen in two of the nine shift sets shown here.

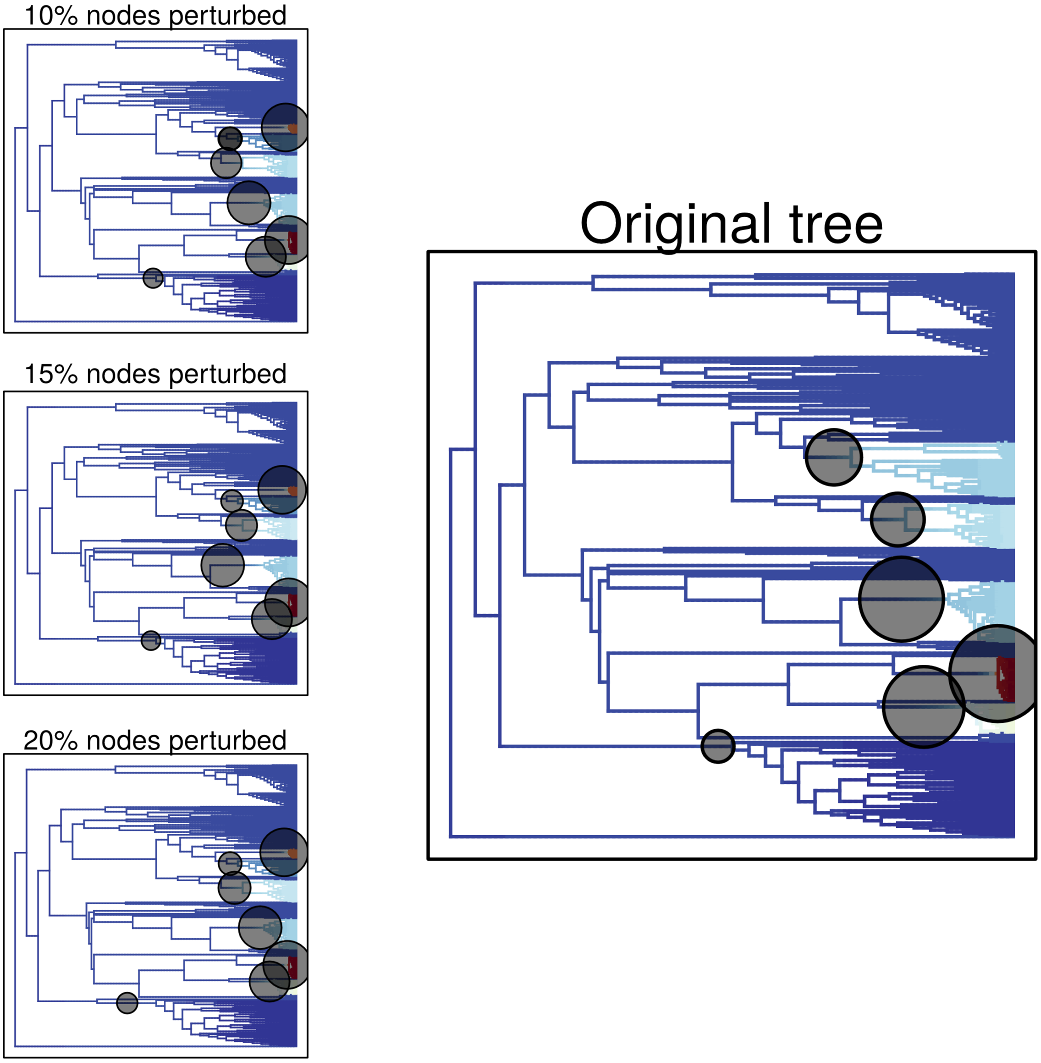

Supplementary Figure 3. Most likely diversification shift patterns observed when calculating diversification rates using trees with minor perturbations in node dates. Three additional trees were calculated with 10, 15 and 20% of dates randomly moved to either a parent or child node (left). The diversification rates show remarkable resilience to this when compared with the unperturbed tree (right), with an additional significant rate shift in the Coenobitidae.

Supplementary Tables

Ref. No	Reference details
1	Ahyong and O'Meally. "Phylogeny of the Decapoda reptantia: Resolution using three molecular loci and morphology". In: Raffles Bulletin of Zoology (2004), 673–693.
2	Ahyong, Schnabel, and Maas. "Anomuran phylogeny: New insights from molecular data". In: Decapod crustacean phylogenetics (2009). 399-414
3	Boyko and Harvey. "Phylogenetic systematics and biogeography of the sand crab families Albuneidae and Blepharipodidae Crustacea: Anomura: Hippoidea)". In: Invertebrate Systematics (2009), 1–18.
4	Bracken et al. "The Decapod Tree of Life: Compiling the Data and Moving toward a Consensus of Decapod Evolution". In: Arthropod Systematics and Phylogeny 67 (2009), 99–116.
5	Cabezas, Macpherson, and Machordom. "Morphological and molecular description of new species of squat lobster (Crustacea: Decapoda: Galatheidae) from the Solomon and Fiji Islands (South-West Pacific)". In: Zoological Journal of the Linnean Society(2009), 465–493.
6	Creasey et al. "Genetic and morphometric comparisons of squat lobster, Munidopsis scobina (Decapoda : Anomura : Galatheidae) populations, with notes on the phylogeny of the genus Munidopsis". In: Deep-sea Research Part Ii-topical Studies In Oceanography (2000), 87–118.
7	Cunningham, Blackstone, and Buss. "Evolution of King Crabs From Hermit- crab Ancestors". In: Nature (1992), 539–542.
8	Dixon, Ahyong, and Schram. "A new hypothesis of decapod phylogeny". In: Crustaceana (2003), 935–975.
9	Hall and Thatje. "Global bottlenecks in the distribution of marine Crustacea: temperature constraints in the family Lithodidae". In: Journal of Biogeography (2009), 2125–2135.
10	Hiller and Werding. "Redescription of Petrolisthes edwardsii (de Saussure) and description of a new, sibling species from the eastern Pacific based on different colour, morphology and genetic identity (Crustacea: Anomura : Porcellanidae)". In: Organisms Diversity & Evolution (2007), 181–194.
11	Hiller et al. "The Petrolisthes galathinus complex: Species boundaries based on color pattern, morphology and molecules, and evolutionary interrelationships between this complex and other Porcellanidae (Crustacea: Decapoda : Anomura)". In: Molecular Phylogenetics and Evolution (2006), 547–569.
12	Hirose, Osawa, and Hirose. "DNA barcoding of hermit crabs of genus Clibanarius Dana, 1852 (Anomura: Diogenidae) in the Ryukyu Islands, southwestern Japan". In: Zootaxa (2010), 59–66.
13	Jones and Macpherson. "Molecular phylogeny of the east pacific squat lobsters of the genus Munidopsis (Decapoda : Galatheidae) with the descriptions of seven new species". In: Journal of Crustacean Biology (2007), 477–501.
14	Lemaitre, Mclaughlin, and Sorhannus. "Phylogenetic relationships within the Pylochelidae (Decapoda: Anomura: Paguroidea): A cladistic analysis based on morphological characters". In: Zootaxa (2009), 1–14.

15	Machordom and Macpherson. “Rapid radiation and cryptic speciation in squat lobsters of the genus Munida (Crustacea, Decapoda) and related genera in the South West Pacific: molecular and morphological evidence”. In: Molecular Phylogenetics and Evolution (2004), 259–279.
16	Macpherson, Jones, and Segonzac. “A new squat lobster family of Galatheoidea (Crustacea, Decapoda, Anomura) from the hydrothermal vents of the Pacific-Antarctic Ridge”. In: Zoosystema (2005), 709–723.
17	Macpherson and Machordom. “Phylogenetic Relationships of Species of Raymunida (Decapoda: Galatheidae) Based on Morphology and Mitochondrial Cytochrome Oxidase Sequences, with the Recognition of Four New Species”. In: Journal of Crustacean Biology (2001), 696–714.
18	Malay and Paulay. “Peripatric Speciation Drives Diversification and Distributional Pattern of Reef Hermit Crabs (decapoda: Diogenidae: Calcinus)”. In: Evolution (2010), 634–662.
19	Mantelatto et al. “Does Petrolisthes armatus (Anomura, Porcellanidae) form a Species Complex or Are We Dealing with Just One Widely Distributed Species?” In: Zoological Studies (2011), 372–384.
20	Mantelatto et al. “Molecular analysis of the taxonomic and distributional status for the hermit crab genera Loxopagurus Forest, 1964 and Isocheles Stimpson, 1858 (Decapoda, Anomura, Diogenidae)”. In: Zoosystema (2006), 495–506.
21	Mantelatto et al. “Taxonomic re-examination of the hermit crab species Pagurus forceps and Pagurus comptus (Decapoda: Paguridae) by molecular analysis”. In: Zootaxa (2009), 20–32.
22	Martin and Abele. “Phylogenetic-relationships of the Genus Aegla (decapoda, Anomura, Aeglidae), With Comments On Anomuran Phylogeny”. In: Journal of Crustacean Biology (1986), 576–616.
23	Mclaughlin, Lemaitre, and Sorhannus. “Hermit crab phylogeny: A reappraisal and its ”fall-out””. In: Journal of Crustacean Biology (2007), 97–115.
24	Morrison et al. “Mitochondrial gene rearrangements confirm the parallel evolution of the crab-like form”. In: Proceedings of the Royal Society of London Series B-biological Sciences (2002), 345–350.
25	Perez-Losada et al. “Conservation Assessment of Southern South American Freshwater Ecoregions on the Basis of the Distribution and Genetic Diversity of Crabs from the Genus Aegla”. In: Conservation Biology (2009), 692–702.
26	Perez-Losada et al. “Molecular systematics and biogeography of the southern South American freshwater ”crabs” Aegla (Decapoda : Anomura : Aeglidae) using multiple heuristic tree search approaches”. In: Systematic Biology (2004), 767–780.
27	Perez-Losada et al. “Phylogenetic position of the freshwater Anomuran family Aeglidae”. In: Journal of Crustacean Biology (2002), 670–676.
28	Perez-Losada et al. “Phylogenetic relationships among the species of Aegla (Anomura : Aeglidae) freshwater crabs from Chile”. In: Journal of Crustacean Biology (2002), 304–313.
29	Porter, Perez-Losada, and Crandall. “Model-based multi-locus estimation of decapod phylogeny and divergence times.” In: Molecular Phylogenetics and Evolution (2005), 355–369.
30	Poupin and Malay. “Identification of a Ciliopagurus strigatus (Herbst, 1804) species-complex, with description of a new species from French Polynesia (Crustacea, Decapoda, Anomura, Diogenidae)”. In: Zoosystema (2009), 209–232.
31	Richter, & Scholtz, and Scholtz. “Phylogeny of the Anomala (Crustacea, Decapoda, Reptantia) based on the ossicles of the foregut”. In: Zoologischer Anzeiger - A Journal of Comparative Zoology

	(2011), 316–342.
32	Schnabel, Ahyong, and Maas. “Galatheoidea are not monophyletic - Molecular and morphological phylogeny of the squat lobsters (Decapoda: Anomura) with recognition of a new superfamily”. In: Molecular Phylogenetics and Evolution (2011), 157–168.
33	Schram. “Phylogeny of decapods: Moving towards a consensus”. In: Hydrobiologia (2001), 1–20.
34	Schram and Dixon. “Decapod phylogeny: addition of fossil evidence to a robust morphological cladistic data set”. In: Bulletin of the Mizunami Fossil Museum (2004), 1–19.
35	Stillman and Reeb. “Molecular Phylogeny of Eastern Pacific Porcelain Crabs, Genera Petrolisthes and Pachycheles, Based on the mtDNA 16S rDNA Sequence: Phylogeographic and Systematic Implications”. In: Molecular Phylogenetics and Evolution (2001), 236–245.
36	Tirelli et al. “Description of the male reproductive system of Paguristes eremita (Anomura, Diogenidae) and its placement in a phylogeny of diogenid species based on spermatozoal and spermatophore ultrastructure”. In: Zoologischer Anzeiger (2010), 299–312.
37	Tirelli et al. “Reproductive biology of Mediterranean hermit crabs: Fine structure of spermatophores and spermatozoa of Diogenes pugilator (Decapoda : Anomura) and its bearing on a sperm phylogeny of Diogenidae”. In: Journal of Crustacean Biology (2008), 534–542.
38	Tsang et al. “Hermit to King, or Hermit to All: Multiple Transitions to Crab-like Forms from Hermit Crab Ancestors”. In: Systematic Biology (2011), 616–629.
39	Tsang et al. “Phylogeny of Decapoda using two nuclear protein-coding genes: Origin and evolution of the Reptantia”. In: Molecular Phylogenetics and Evolution (2008), 359–368.
40	Tudge. “Phylogeny of the Anomura (Decapoda, Crustacea): Spermatozoa and spermatophore morphological evidence”. In: Contributions To Zoology (1997), 125–141.
41	Werding, Hiller, and Misof. “Evidence of paraphyly in the neotropical Porcellanid genus Neopisosoma (Crustacea : Anomura : Porcellanidae) based on molecular characters”. In: Hydrobiologia pp(pp2001), 105–110.

Supplementary Table 1. References used as source trees in supertree construction. For each source all relevant trees were encoded in Nexus format with in the STK[4] and included in supplementary data file.

Cancellocheles sculptipes
Dardanus setifer
Clibanarius albidigitus
Clibanarius antillensis
Forestocheles perplexus
Gastroptychus rogeri
Janetogalathea californiensis
Mixtopagurus paradoxus
Munida albiapicula
Munida quadrispina
Munida sp.
Munida valida
Munidopsis segonzaci
Munidopsis sp.
Paguristes bakeri
Paguristes seminudus
Pagurus ochotensis
Pagurus spp.

Paramunida scabra
Paramunida tricarinata
Parapylocheles scorpio
Pleuroncodes planipes
Probeebei mirabilis
Pylocheles inarmatus
Sympagurus sp.
Uroptychodes grandirostris

Supplementary Table 2. List of all "rogue" taxa removed from final supertree. Each of the taxa listed above had poor placement in the final supertree not supported by any source tree and hence they were removed before any subsequent analysis.

Supplementary References

1 PAUP*: phylogenetic analysis using parsimony. Sinauer Associates, Sunderland, Massachusetts (2003).

2 Bell, M. A., Lloyd, G. T. & Smith, A. strap: an R package for plotting phylogenies against stratigraphy and assessing their stratigraphic congruence. *Palaeontology* **58**, 379-389, doi:10.1111/pala.12142 (2015).

3 Rabosky, D. L. Automatic Detection of Key Innovations, Rate Shifts, and Diversity-Dependence on Phylogenetic Trees. *PLoS ONE* **9**, e89543, doi:10.1371/journal.pone.0089543 (2014).

4 Hill, J. & Davis, K. The Supertree Toolkit 2: a new and improved software package with a Graphical User Interface for supertree construction. *Biodiversity Data Journal* **2**, e1053, doi:10.3897/BDJ.2.e1053 (2014).
